# Different Patient Subgroup Different Maintenance, Proteasome Inhibitors or Immunomodulators Maintenance for Newly Diagnosed Multiple Myeloma: A 7-Year Single-Center Data in China

**DOI:** 10.3389/fonc.2021.665217

**Published:** 2021-06-14

**Authors:** Xiaoyan Han, Chunxiang Jin, Gaofeng Zheng, Donghua He, Yi Zhao, Yi Li, Wenjun Wu, Weiyan Zheng, Guoqing Wei, Enfan Zhang, He Huang, Jingsong He, Zhen Cai

**Affiliations:** ^1^Bone Marrow Transplantation Center, Department of Hematology, The First Affiliated Hospital, School of Medicine, Zhejiang University, Hangzhou, China; ^2^Institute of Hematology, Zhejiang University, Hangzhou, China

**Keywords:** multiple myeloma, maintenance, proteasome inhibitors, immunomodulators, optimal maintenance duration

## Abstract

**Introduction:**

We analyzed different patient subgroups to determine optimal maintenance therapy in newly diagnosed multiple myeloma (NDMM) patients.

**Methods:**

A total of 226 NDMM patients in our center were included in the study. The characteristics, survival, and adverse reactions were compared among patients who received maintenance therapy or not, and patients who received proteasome inhibitors (PIs) or immunomodulators (IMiDs) maintenance. The survival of different maintenance durations of bortezomib-based regimens was also analyzed.

**Results:**

The maintenance therapy not only upgraded more patient responses (34.3 *vs* 13.3%, *P* = 0.006), but also significantly prolonged their progression-free survival (PFS) (median PFS: 41.1 *vs* 10.5 months, *P* < 0.001) and overall survival (OS) (median OS: not reached *vs* 38.6 months, *P* < 0.001). Compared with IMiDs, the PFS (median PFS: 43.7 *vs* 38.5 months, *P* = 0.034) and OS (median OS: not reached *vs* 78.5 months, *P* = 0.041) were both enhanced by PIs maintenance. Patients younger than 65 years who received PIs had a significantly prolonged OS (*P* = 0.032). Patients achieving only a partial response (PR) after induction and consolidation therapy had significantly longer PFS and OS after PIs maintenance compared to IMiDs (*P* = 0.007, 0.002). High-risk patients (ISS 2–3, DS 2–3, and RISS 2–3) given PIs maintenance benefit from a prolonged PFS (*P* = 0.002, 0.02, 0.06) and OS (*P* = 0.059, 0.047, 0.044, respectively) compared with IMiDs therapy. OS was significantly prolonged in patients who received ≥ 12 months of bortezomib-based maintenance therapy compared to those who were treated for < 12 months (*P* < 0.001), but no difference was observed in OS between patients who received 12 to 24 or ≥ 24 months of bortezomib-based maintenance therapy (*P* = 0.292).

**Conclusion:**

PIs maintenance was superior to IMiDs in overall PFS and OS. The beneficial effect was most evident in patients achieving PR after induction and consolidation therapy, and in high-risk patients. Moreover, younger patients also benefited from PIs maintenance with an increased OS. A bortezomib-based maintenance therapy duration of 12 to 24 months after induction and consolidation therapy produced satisfactory OS.

## Introduction

Multiple myeloma (MM), a clonal plasma cell neoplasm characterized by monoclonal immunoglobulin production, is the second most common hematological malignancy and accounts for about 1% to 2% of all cancers (1). In the past two decades, autologous stem cell transplantation (ASCT) and the development of novel drugs have significantly improved the outcomes for MM patients. However, most patients inevitably experience disease progression or relapse, so maintenance therapy has become a necessary means to improve and sustain the depth of the response, as well as survival improvement (2, 3). The bortezomib-based regimens are widely used in induction therapy (4–6). Yet its experience in maintenance therapy is still limited. Therefore, we have summarized the 7-year follow-up data of MM patients in our center and compared proteasome inhibitors (PIs) bortezomib-based regimens with the immunomodulators (IMiDs), thalidomide and lenalidomide, in maintenance therapy. The aim was to clarify their roles in the upgrade of responses, survival improvement and to evaluate adverse reactions. Subgroup analysis was employed to illuminate the most appropriate maintenance therapy approach for corresponding patient subgroups.

## Materials and Methods

### Patients

Newly diagnosed multiple myeloma (NDMM) patients who had achieved at least a partial response (PR) after induction and consolidation therapy with bortezomib-based regimens at the First Affiliated Hospital of Zhejiang University Medical College from May 23, 2013, to December 13, 2018, were evaluated for inclusion in our retrospective study. All patients were followed up to assess mortality and survival until October 1, 2020. Their demographics, disease characteristics, and treatment regimens were extracted from electronic medical records after approval was granted by the appropriate review boards. Patients were stratified according to the Durie Salmon (DS) stage, International Staging System (ISS) disease stage, and a revised-ISS (R-ISS) stage at diagnosis. Due to the lack of FISH data, 20 patients in the maintenance group (including 15 patients in the PIs subgroup and five patients in the IMiDs subgroup) and two patients in the no maintenance group could not be stratified by RISS staging.

### Treatment Regimens

All patients received bortezomib-based regimens as induction therapy, including PD (bortezomib and dexamethasone), PCD (bortezomib, cyclophosphamide, and dexamethasone), PAD (bortezomib, doxorubicin, and dexamethasone), PTD (bortezomib, thalidomide, and dexamethasone), and VRD (bortezomib, lenalidomide, and dexamethasone). All patients received subcutaneous bortezomib 1.3 mg/m^2^; young patients (< 65 years old) received a 28-day course of treatment on days 1, 4, 8, and 11. Older patients (≥ 65 years old) received a 35-day course of treatment on days 1, 8, 15, and 22. Dexamethasone 20 mg/day was administered intravenously following bortezomib on days 1–2, 4–5, 8–9, and 11–12 (or days 1–2, 8–9, 15–16, and 22–23). Similarly, doxorubicin (10 mg/m^2^) and cyclophosphamide (200 mg/m^2^) were given intravenously on days 1, 4, 8, and 11 (or days 1, 8, 15, and 22). Thalidomide (100 mg/day) and lenalidomide (10–25 mg/day) were taken orally during the entire treatment cycle.

After three to four cycles of induction therapy, ASCT was implemented in a number of patients eligible for transplantation, according to their age, general state, and willingness. Other patients not eligible for transplantation continued to receive two to four courses of consolidation therapy, which was basically the same as the induction regimens.

After three to four cycles of induction and consolidation therapy with or without ASCT, the majority of patients received maintenance therapy. A large proportion of patients received PIs -based regimens such as PD, PCD, PAD, PTD, VRD for maintenance. During maintenance, all patients received bortezomib-based regimens with a 3-month cycle on days 1, 8, 15, and 22, the doses were basically as same as the induction therapy unless individual patients are intolerant because of severe adverse reactions. The other group of patients received immunomodulators as maintenance therapy, such as T (thalidomide), R (lenalidomide), TD (thalidomide and dexamethasone), and RD (lenalidomide and dexamethasone), thalidomide was taken everyday, and lenalidomide was taken on days 1 to 21 of 28-day cycles.

### Efficacy and Safety Evaluation

Outcome measures included the response to treatment, overall survival (OS), and progression-free survival (PFS). The response to treatment was defined as the International Myeloma Working Group uniform response criteria, including partial response (PR), very good partial response (VGPR), and complete response (CR) (7). To assess adverse reactions, we adopted the National Cancer Institute Common Toxicity Criteria for Adverse Events, version 5.0.

### Statistical Analysis

Statistical analyses were carried out using SPSS 21.0 software (SPSS, Chicago, IL, US). Baseline characteristics were evaluated using descriptive statistical analysis: frequency distributions (n, %) are presented for categorical variables and compared using the chi-squared test. The median (range) is presented for continuous variables and compared using a nonparametric T-test, only age was continuous variable in our study. PFS and OS analyses were performed using the Kaplan-Meier method, and the log-rank test was used to analyze the differences between survival curves. A value of *P* < 0.05 was taken to indicate statistical significance, and all tests were two-sided.

## Results

### Maintenance *vs* No Maintenance

#### Characteristics of Patients

The clinical data and biological characteristics of 181 patients who received maintenance therapy and 45 patients who did not are summarized in **Table 1**. Significant differences were found in ASCT. The proportion of patients who adopted ASCT was higher in the maintenance group (17.1 *vs* 2.3%). Other baseline characteristics were basically similar.

**Table 1:** Baseline characteristics of NDMM patients with or without maintenance therapy.

	Maintenance, N = 181	No maintenance, N = 45	*P*-value
Characteristics	N (%)	N (%)	
Age (years)			
Median (range)	62 (39–84)	61 (44–77)	0.790
Gender			0.129
Male	98 (54.1)	30 (66.7)	
Female	83 (45.9)	15 (33.3)	
Type of myeloma			0.445
IgA	48 (26.5)	12 (26.7)	
IgD	11 (6.1)	3 (6.7)	
IgG	75 (41.4)	24 (53.3)	
Light chain	46 (25.4)	6 (13.3)	
Biphenotypic	1 (0.6)	0 (0)	
ISS stage			0.962
1	68 (37.6)	16 (35.6)	
2	56 (30.9)	14 (31.1)	
3	57 (31.5)	15 (33.3)	
RISS^a^ (N = 204)			0.199
1	22 (13.7)	2 (4.7)	
2	103 (64.0)	28 (65.1)	
3	36 (22.4)	13 (30.2)	
Durie-Salmon stage			0.360
1A+1B	17 (9.4)	7 (15.6)	
2A+2B	22 (12.2)	7 (15.6)	
3A+3B	142 (78.5)	31 (68.9)	
ASCT			0.011
Yes	31 (17.1)	1 (2.3)	
No	150 (82.9)	44 (97.7)	
Response after induction			0.267
PR	72 (39.8)	22 (48.9)	
≥ VGPR	109 (60.2)	23 (51.1)	
Induction therapy			0.065
PAD	27 (14.9)	6 (13.3)	
PCD	122 (67.4)	28 (62.2)	
PD	25 (13.8)	4 (8.9)	
PTD	5 (2.8)	5 (11.1)	
VRD	2 (1.1)	2 (4.4)	

a Due to a lack of FISH data, 20 patients of the maintenance group and 2 patients of the no maintenance group could not be stratified by RISS staging.

NDMM, newly diagnosed multiple myeloma; ISS, International Staging System; R-ISS, revised International Staging System; ASCT, autologous hematopoietic stem cell transplantation; PR, partial response; VGPR, very good partial response; PAD, bortezomib, doxorubicin and dexamethasone; PCD, bortezomib, cyclophosphamide and dexamethasone; PD, bortezomib and dexamethasone; PTD, bortezomib, thalidomide and dexamethasone. VRD, bortezomib, lenalidomide, and dexamethasone.

#### Survival

**Figure 1:**
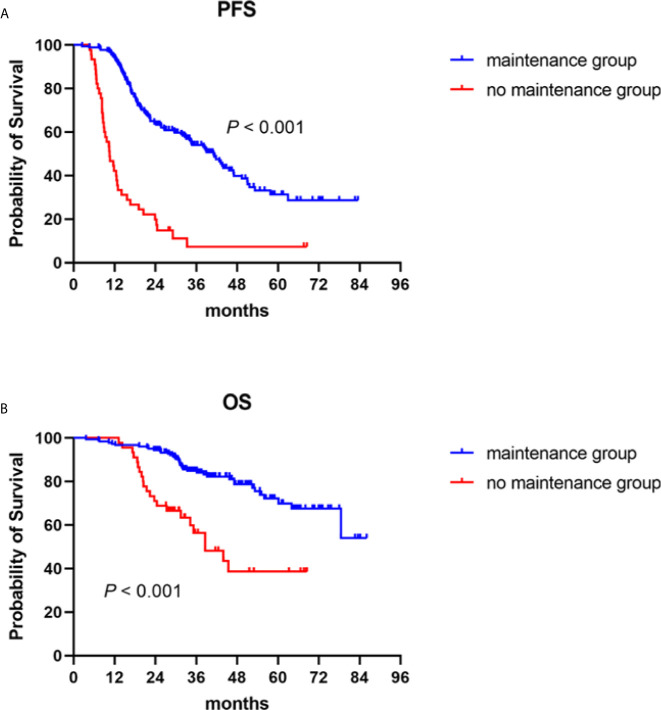
Progression-free (PFS) and overall survival (OS) in NDMM patients with or without maintenance therapy. Kaplan–Meier curves are shown for **(A)** PFS and **(B)** OS.

The median follow-up duration for all patients was 36.9 (3.6–86.0) months, the median PFS 41.1 (95% CI: 34.5–47.7) months for the patients who received maintenance treatments, and 10.5 (95% CI: 8.0–13.1) months for those in the no maintenance group (*P* < 0.001) **(** **Figure 1A** **)**. The median OS of the patients who received maintenance therapy was not reached. The median OS of patients who were not given maintenance therapy was 38.6 (95% CI: 27.0–50.2) months. Survival was distinctly prolonged for patients who received maintenance therapy (*P* < 0.001) **(** **Figure 1B** **).**

### Response

During maintenance therapy, the upgrade of response from PR to at least VGPR was more common in the maintenance group (34.3 *vs* 13.3%, *P* = 0.006). The best responses after maintenance therapy, the PR rate was 17.7% and at least a VGPR rate of 82.3% in the maintenance group *vs* 42.9% and 57.8% in the no maintenance group (*P* < 0.001).

### Proteasome Inhibitors *vs* Immunomodulators

#### Characteristics of Patients

The baseline characteristics of 181 patients who received maintenance therapy, including 127 with PIs and 54 with IMiDs, are presented in **Table 2**. No significant statistical differences were found between the two groups of variables. In the PIs group, there were 7 (5.5%) patients with PAD, 93 (73.2%) with PCD, 21 (16.5%) with PD, and 6 (4.8%) with VRD maintenance therapy, respectively. In the IMiDs group, there were 5 (9.3%) patients with R, 28 (51.9%) with RD, 19 (35.2%) with T and 2 (3.7%) with TD maintenance therapy.

**Table 2:** Baseline characteristics of NDMM patients who received maintenance therapy of proteasome inhibitors or immunomodulators.

	PIs, N = 127	IMiDs, N = 54	*P*-value
Characteristics	N (%)	N (%)	
Age (years)			
Median (range)	62 (41–82)	63 (39–82)	0.153
<65 years	83 (65.4)	30 (55.6)	0.213
≥65 years	44 (34.6)	24 (44.4)	
Gender			0.120
Male	64 (50.4)	34 (63.0)	
Female	63 (49.6)	20 (37.0)	
Type of myeloma			0.219
IgA	35 (27.6)	13 (24.1)	
IgD	8 (6.3)	3 (5.6)	
IgG	46 (36.2)	29 (53.7)	
Light chain	37 (29.1)	9 (16.7)	
Biphenotypic	1 (0.8)	0 (0)	
ISS stage			0.745
1	48 (37.8)	20 (37.0)	
2	41 (32.3)	15 (27.8)	
3	38 (29.9)	19 (35.2)	
^a^RISS stage (N = 161)			0.684
1	14 (12.5)	8 (16.3)	
2	74 (66.1)	29 (59.2)	
3	24 (21.4)	12 (24.5)	
Durie-Salmon stage			0.229
1A+1B	12 (9.4)	5 (9.3)	
2A+2B	12 (9.4)	10 (18.5)	
3A+3B	103 (81.1)	39 (72.2)	
Induction therapy			0.069
PAD	19 (15.0)	8 (14.8)	
PCD	85 (66.9)	37 (68.5)	
PD	21 (16.5)	4 (7.4)	
PTD	1 (0.8)	4 (7.4)	
VRD	1 (0.8)	1 (1.9)	
Response after induction			0.134
PR	48 (37.8)	24 (44.4)	
VGPR	22 (17.3)	14 (25.9)	
CR	57 (44.9)	16 (29.6)	

a Due to a lack of FISH data, 15 patients in the PIs subgroup and 5 patients in the IMiDs subgroup could not be stratified by RISS staging.

PIs, proteasome inhibitors; IMiDs, immunomodulators.

#### Survival

**Figure 2:**
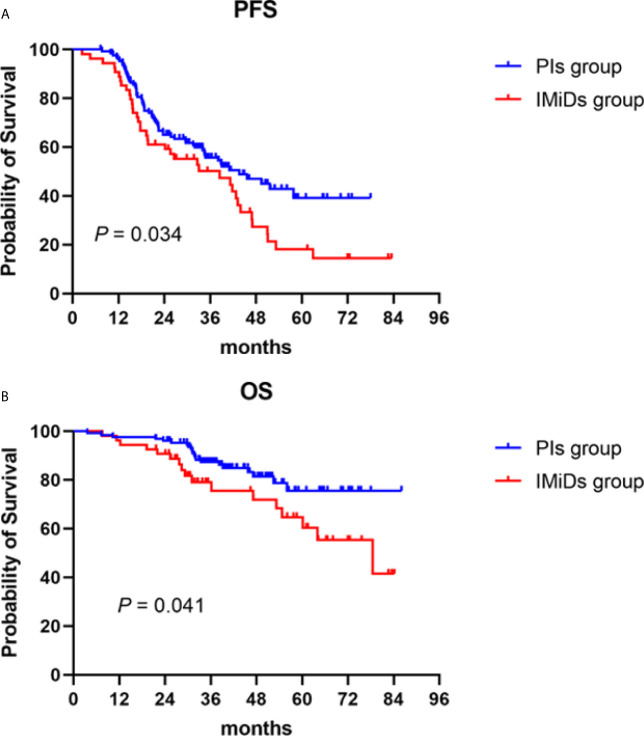
Progression-free (PFS) and overall survival (OS) in NDMM patients who received PIs or IMiDs maintenance therapy. Kaplan–Meier curves are shown for **(A)** PFS and **(B)** OS. PIs, proteasome inhibitors; IMiDs, immunomodulators.

The median follow-up of the total maintenance patients was 39.2 (3.6–86.0) months. During the follow-up, 62 patients (48.8%) and 38 patients (70.4%) had disease recurrence or progression in the PIs and IMiDs groups, respectively, with a median PFS of 43.7 (95% CI: 30.3–57.1) months *vs* 38.5 (95% CI: 19.1–58.0) months (*P* = 0.034) **(** **Figure 2A** **)**. As for the overall survival, 21 patients (16.5%) and 17 patients (31.5%) died during the follow-up, with a median OS not reached in the PIs group *vs* 78.5 (50.1–106.9) months in the IMiDs group (*P* = 0.041) **(** **Figure 2B** **)**.

#### Subgroup Analysis

##### Age and Creatinine Levels

For patients younger than 65 years old, maintenance therapy with PIs significantly prolonged OS (*P* = 0.032), with a 5-year OS of 81.5 *vs* 66.1%, respectively. No statistical difference was found in PFS between the two groups, with a 3-year PFS of 56.6 *vs* 55.0%. As for patients aged 65 years and older, there was no significant difference in neither PFS nor OS between the two groups. For renal functions, OS benefit was observed in the PIs group in patients with baseline serum creatinine < 2 mg/dL, with a 5-year OS of 76.8 *vs* 64.9% (*P* = 0.036). In patients with normal renal functions, PI maintenance therapy appeared to improve PFS compared with the IMiDs group, with a 3-year PFS of 58.4 *vs* 52.0% (*P* = 0.070), although statistical significance was not reached. In patients whose baseline serum creatinine was ≥ 2 mg/dl, there was no difference in PFS and OS between the two maintenance therapy options **(** **Table 3** **)**.

**Table 3:** Subgroup analysis of NDMM patients who received maintenance therapy of PIs or IMiDs.

Median Survival (m) (95% CI)	Progression-free survival		*P*-value	Overall survival		*P*-value
	PIs	IMiDs		PIs	IMiDs	
Age < 65 years (n = 113)	45.7 (30.9–60.5)	41.8 (22.1–61.5)	0.205	NR	78.5 (54.9–102.0)	0.032
3-PFS (5-OS) (%)	56.6	55.0		81.5	66.1	
Age ≥ 65 years (n = 68)	43.7 (30.0–57.3)	26.5 (13.8–39.2)	0.115	NR	NR	0.511
3-PFS (5-OS) (%)	54.1	44.4		61.6	62.1	
Creatinine						
<2 mg/dl (N = 153)	43.7 (31.5–55.9)	38.5 (22.2–54.8)	0.070	NR	78.5 (49.8–107.1)	0.036
3-PFS (5- OS) (%)	58.4	52.0		76.8	64.9	
≥2 mg/dl (N = 28)	31.5 (11.4–51.6)	17.7 (13.5–22.0)	0.141	NR	NR	0.570
3-PFS (5-OS) (%)	44.9	0		70.8	66.7	
Response after induction						
PR (N = 72)	34.0 (19.0–49.0)	17.1 (2.7–21.6)	0.007	NR	47.1 (none)	0.002
3-PFS (5-OS) (%)	44.8	25.0		77.9	45.1	
≥ VGPR (N = 109)	51.6 (36.8–66.4)	46.9 (35.4–58.4)	0.831	NR	NR	0.826
3-PFS (5-OS) (%)	62.2	70.3		78.9	80.5	
Non-high risk						
ISS1(N = 68)	39.0 (25.6–52.4)	51.0 (40.9–61.0)	0.848	NR	NR	0.320
3-PFS (5-OS) (%)	53.8	64.6		81.5	76.5	
DS 1 (N = 17)	29.6 (10.5–48.7)	NR	0.901	NR	NR	0.637
3-PFS (5-OS) (%)	48.6	60.0		88.9	100.0	
RISS 1 (N = 22)	38.9 (24.0–53.7)	19.7 (6.0–33.4)	0.107	NR	47.2 (19.5–74.9)	0.057
3-PFS (5-OS) (%)	55.6	37.5		73.8	50.0	
High Risk						
ISS 2–3 (N = 113)	43.7 (28.9–58.4)	26.5 (11.7–41.4)	0.002	NR	64.0 (41.6–86.4)	0.059
3-PFS (5-OS) (%)	57.0	40.3		71.4	55.2	
DS 2–3 (N = 164)	45.7 (34.2–57.2)	33.1 (16.1–50.0)	0.020	NR	78.5 (52.3–104.6)	0.047
3-PFS (5-OS) (%)	56.5	49.5		73.9	62.9	
RISS 2–3 (N = 139)	39.0 (29.6–48.5)	33.1 (15.8–50.4)	0.060	NR	78.5 (51.8–105.1)	0.044
3-PFS (5-OS) (%)	52.4	48.6		75.6	62.6	

m, months; 3-PFS, 3 years of PFS; 5-OS, 5 years of OS; NR, not reached.

##### Responses After Induction and Consolidation Therapy

Patients achieving only a PR after induction and consolidation therapy had a significantly longer PFS and OS with PIs maintenance therapy compared with IMiDs, with a 3-year-PFS of 44.8 and 25.0% (*P* = 0.007) and a 5-year-OS of 77.9 and 45.1%, respectively (*P* = 0.002). However, in those patients achieving at least VGPR, no difference was found between the 2 groups in PFS or OS **(** **Table 3** **)**.

#### Clinical Stages

Patients were stratified according to their clinical stages. Patients in ISS 1, DS 1, and RISS 1 were classified as non–high-risk; the other patients in ISS 2–3, DS 2–3, and RISS 2–3 were classified as high-risk. Overall, high-risk patients who received PIs maintenance therapy had improved survival times. The 3-year PFS of high-risk patients who were given PIs or IMiDs maintenance therapy was 57.0 *vs* 40.3%, 56.5 *vs* 49.5%, and 52.4 *vs* 48.6%, respectively (*P* = 0.002, 0.02, 0.06). The 5-year OS of high-risk patients who received PIs or IMiDs maintenance therapy was 71.4 *vs* 55.2%, 73.9 *vs* 62.9% and 75.6 *vs* 62.6%, respectively (*P* = 0.059, 0.047, 0.044). In non–high-risk patients, no difference was found in PFS or OS between the two maintenance therapy options **(** **Table 3** **)**.

#### Adverse Reactions

During maintenance therapy, there were no significant statistical differences in adverse reactions between the two maintenance therapy options. The incidence of second primary malignancies (SPMs) was slightly higher in the IMiDs group (0 *vs* 3.7%, *P* = 0.088). The most common hematological adverse events are thrombocytopenia (5.5 *vs* 7.4%) and neutropenia (4.7 *vs* 3.7%). For non-hematological adverse events, the most frequent were peripheral neuropathy (23.0 *vs* 33.3%) and infection (22.0 *vs* 25.9%). A summary of the results is presented in **Table 4**.

**Table 4:** Analysis of adverse reactions produced by PIs and IMiDs maintenance therapy.

Adverse events, n (%)	PIs	IMiDs	*P*-value
Hematologic events (3/4 grade)			
Thrombocytopenia	7 (5.5%)	4 (7.4%)	0.882
Neutropenia	6 (4.7%)	2(3.7%)	1.000
Anemia	1 (0.8%)	2 (3.7%)	0.441
Non-hematologic events (all grades)			
Peripheral neuropathy	29 (23.0%)	18 (33.3%)	0.149
Infection	28 (22.0%)	14 (25.9%)	0.572
Fatigue	16 (12.6%)	10 (18.5%)	0.299
Herpes zoster	9 (7.1%)	3 (5.6%)	0.958
Constipation	6 (4.7%)	6 (11.1%)	0.114
Diarrhea	5 (3.9%)	3 (3.2%)	0.929
Second primary malignancies	0 (0%)	2 (3.7%)	0.088

### Treatment Duration of Maintenance Therapy With PIs

**Figure 3:**
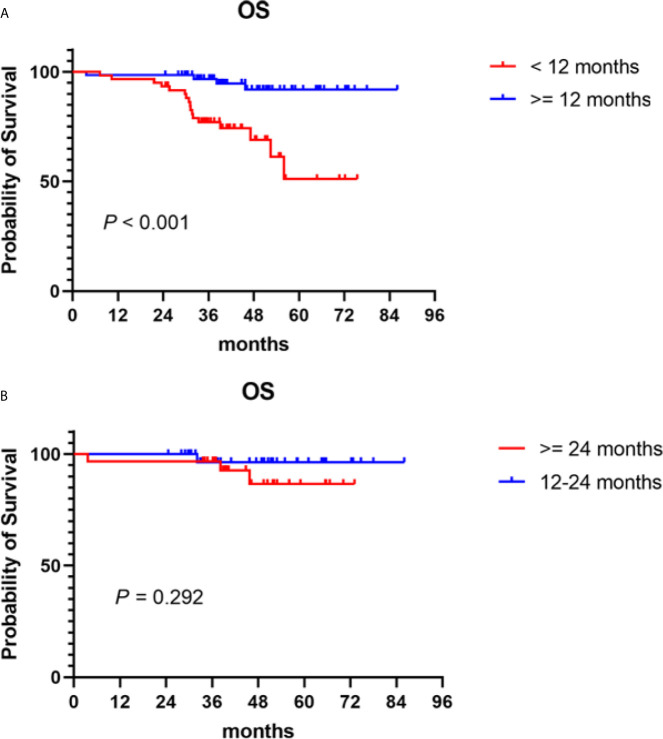
Overall survival (OS) in NDMM patients who received PIs maintenance therapy of different treatment durations. Kaplan–Meier curves are shown for **(A)** patients who received ≥ 12 months of bortezomib-based maintenance therapy *vs* those who were given maintenance therapy for < 12 months; **(B)** patients who received 12–24 months of bortezomib-based maintenance therapy *vs* those given maintenance therapy for ≥ 24 months.

Bortezomib-based regimens have become the main maintenance therapy options in recent years. This paradigm of long-term treatment needs to consider many other factors, such as patients’ quality of life, convenience, and the burden of long-term treatment. Thus, we performed a secondary analysis to establish the optimal treatment duration. The median treatment duration of patients who received bortezomib-based maintenance therapy after induction and consolidation therapy was 12.9 (0.8–45.1) months. PFS improved with increasing treatment duration (*P* < 0.001). OS was significantly prolonged in patients who received ≥ 12 months of bortezomib-based maintenance therapy compared to those given maintenance therapy for < 12 months, with a 5-year OS of 91.9 *vs* 51.1% (*P* < 0.001) **(** **Figure 3A** **)**. However, no difference was found in OS between patients who received 12 to 24 or ≥ 24 months of bortezomib-based maintenance therapy (*P* = 0.292), with a 5-year OS of 96.4 *vs* 86.6% **(** **Figure 3B** **)**.

The relationship between the maintenance treatment course and the outcome was consistent with the above results. The median maintenance cycles completed were 4 (1–14) in patients after induction and consolidation therapy. PFS was clearly improved with an increase in the number of treatment cycles (*P* < 0.001). OS was significantly different in patients with < 4 *vs* ≥ 4 maintenance treatment courses (*P* < 0.001), with a 5-year OS of 42.3 *vs* 92.7%. The OS between patients with 4–9 *vs* ≥ 9 maintenance treatment courses was not statistically significant (*P* = 0.214), with a 5-year OS of 95.5 *vs.* 83.7%.

## Discussion

In the past two decades, with the introduction of new drugs such as proteasome inhibitor bortezomib, and the immunomodulators thalidomide and lenalidomide, the treatment paradigm of NDMM has changed dramatically. Those new drugs have less toxicity than traditional chemotherapy drugs, allowing long-term maintenance to become a treatment paradigm. Maintenance therapy usually refers to administering a course of long-term chemotherapy after induction and consolidation therapy, two typical maintenance paradigms have been implanted in our center: one was to prolong the chemotherapy interval of bortezomib-based regimens with a 3-month cycle, the other was to adopt IMiDs continuous treatment. In the present study, the significance of maintenance treatment not only improved more patients’ responses (34.3 *vs* 13.3%) but also significantly prolonged the patients’ PFS (median PFS: 41.1 *vs* 10.5 months) and OS (median OS: not reached *vs* 38.6 months). We admit that the PFS was shorter compared with values reported in the literature in no maintenance group (8–10). The reasons for the discrepancy may be as follows: first, almost all of our non-maintenance patients did not undergo ASCT. Moreover, the patients did not receive maintenance therapy partly because of their poor physical condition. Our limited sample size may also have had an impact on the results, but the survival benefit of maintenance therapy is beyond reasonable doubt (8–14).

Various maintenance treatment options for NDMM are recommended within consensus guidelines (15–17). The consensus of the Mayo Clinic recommends: immunomodulators maintenance for patients with standard-risk cytogenetics and PI maintenance therapy for patients with high-risk cytogenetics (16). Data from our center revealed that compared with thalidomide and lenalidomide, PFS and OS can both benefit from bortezomib-based regimens (median PFS: 43.7 *vs* 38.5 months; median OS: not reached *vs* 78.5 months), findings consistent with the reported literature (18). It is worth noting that in the phase III HOVON-65/GMMG-HD4 trial, the early research report showed that OS was superior after bortezomib-based regimens for induction and maintenance compared to non–bortezomib-based regimen inductions and thalidomide maintenance therapy (19). However, after long-term follow-up, the OS was no difference between the two study arms, due to the majority of patients having relapsed and had to receive multiple effective post-relapse treatments (20).

To explore further the best maintenance therapy options for patients in the different subgroups, we conducted a more detailed subgroup analysis. In younger patients, the OS was superior in patients who were given bortezomib-based maintenance therapy (median OS: not reached *vs* 78.5 months). Bortezomib does not require dose adjustment in MM patients with renal impairment (21). Moreover, bortezomib-based regimens before and after ASCT can overcome the adverse effects of renal damage on prognosis (19, 20, 22). Our data showed that for patients with baseline serum creatinine < 2 mg/dl, both PFS and OS benefited from bortezomib-based maintenance therapy, whereas in patients with baseline serum creatinine ≥ 2 mg/dl, no statistical difference was observed between the 2 maintenance options. It may be because most of our patients did not undergo ASCT; the bortezomib-based regimens induction and maintenance therapy without ASCT may not have been sufficient to overcome the adverse effects of renal impairment on prognosis. Moreover, due to our limited sample size, we need to interpret this conclusion cautiously and expand the number of patients to verify unequivocally our conclusion in future studies. The response after induction and consolidation was also an important factor in the choice of maintenance treatment options. In patients who achieved only PR after induction and consolidation, the bortezomib-based maintenance therapy significantly prolonged PFS and OS. However, in patients who achieved at least VGPR, there was no statistical difference in PFS and OS between the two groups. Clinic`al trial data have shown that bortezomib consolidation after ASCT only improved the PFS of patients not achieving at least VGPR, had no effect on patients who achieved at least VGPR, and did not prolong OS in both categories of patients (23). Consolidation therapy refers to the utilization of a short course of treatment to reduce the number of residual tumor cells, which can prolong PFS but not OS (18). Consolidation and maintenance therapy play a different role in patients’ outcomes. In high-risk patients (including ISS 2–3, DS 2–3, and RISS 2–3), it is essential to choose bortezomib-based maintenance therapy; PFS and OS were significantly superior to thalidomide or lenalidomide maintenance therapy. However, in the non–high-risk patients, the difference did not reach statistical significance between the two groups. With respect to the adverse reactions, the overall incidence of maintenance therapy was lower than induction therapy for prolonging the chemotherapy interval or simplification of chemotherapy regimens. There were no significant statistical differences between the two maintenance therapy options. In our study, two patients occurred SPMs in the IMiDs group, one had lung cancer and the other had cervical cancer; SPMs were not observed in the PIs group, statistical significance was not reached. A meta-analysis showed that the combination of lenalidomide with oral melphalan increased the incidence of hematological SPMs versus melphalan alone, but did not affect the incidence of solid tumors (24). The long-term results of the phase III HOVON-65/GMMG-HD4 trial also indicated the incidence of SPMs was similar between the thalidomide and bortezomib maintenance groups (20). As for peripheral neuropathy (PN), subcutaneous administration of bortezomib, prolonging chemotherapy interval, and treating PN with acupuncture and vitamin B12, PN can be tolerated in most patients during maintenance.

The NCCN guidelines have added bortezomib-based maintenance therapy as an option for patients with or without ASCT (25). In PI maintenance therapy, the PCD regimen was the most adopted option, due to its greater efficacy, fewer adverse reactions, lower cost, and convenience to administer. It is the first-line treatment for NDMM approved by multiple centers in induction therapy (26–28), which is also widely used in maintenance therapy. The treatment duration usually lasts 2 to 3 years or until the disease progresses. We performed a secondary analysis to determine the optimal treatment duration, the optimal duration that the patient can bear for the best disease relief, but also to reduce the burden of long-term maintenance. Through our analysis, a bortezomib-based maintenance duration lasting 12 to 24 months after induction and consolidation therapy was shown to produce a satisfactory OS, the outcome for maintaining treatment for > 24 months was similar to that of 12 to 24 months. It is equivalent to four to eight courses of maintenance treatment after induction and consolidation treatment, with a 3-month cycle.

The first oral bioavailable PI, ixazomib, was licensed for the treatment of MM in China in 2018. Multiple studies have confirmed its effectiveness and safety (29, 30). Ixazomib has become a category 1 “other recommended” maintenance therapy option for patients eligible for transplantation in the NCCN guidelines (25). Ixazomib is likely to play an important role in future maintenance regimens. Various studies are ongoing to explore the best maintenance therapy options according to different patient subgroups and to optimize individual patient outcomes. At the same time, the studies may be able to provide different maintenance options. In the future, in the maintenance paradigm, not only efficacy and adverse effects should be considered but also the quality of the lives of the patients, convenience, compliance with long-term treatment, and not least the financial burden. In addition, the role of minimal residual disease (MRD) in guiding maintenance treatment decisions has been receiving increasing attention.

## Conclusion

In our study, we demonstrated the efficacy of bortezomib-based regimens as maintenance therapy. We put forward suggestions for optimal maintenance therapy according to different patient subgroups and provided a reference for the optimal duration of maintenance therapy. Due to sample size limitations, our conclusions need to be interpreted carefully. At the same time, we will continue focusing on maintenance therapy in future research with larger sample size, longer follow-up, and more maintenance therapy options.

## Data Availability Statement

The raw data supporting the conclusions of this article will be made available by the authors, without undue reservation.

## Ethics Statement

Written informed consent was obtained from the individual(s) for the publication of any potentially identifiable images or data included in this article.

## Author Contributions

XH and JH contributed to the study design and provided the data. CJ wrote the manuscript and reviewed the literature. GZ, YL, WW, WZ, GW, and HH reviewed and wrote parts of the manuscript. EZ reviewed the manuscript and provided images. ZC supervised and formulated the manuscript, wrote the final manuscript and conducted a review of the literature. All authors contributed to the article and approved the submitted version.

## Funding

This study was supported by the National Natural Science Foundation of China (grant number 81800201), and the Natural Science Foundation of Zhejiang Province (grant number LY17H080001).

## Conflict of Interest

The authors declare that the research was conducted in the absence of any commercial or financial relationships that could be construed as a potential conflict of interest.
