# Corrigendum: Different Patient Subgroup Different Maintenance, Proteasome Inhibitors or Immunomodulators Maintenance for Newly Diagnosed Multiple Myeloma: A 7-Year Single-Center Data in China

**DOI:** 10.3389/fonc.2021.740715

**Published:** 2021-09-02

**Authors:** Xiaoyan Han, Chunxiang Jin, Gaofeng Zheng, Donghua He, Yi Zhao, Yi Li, Wenjun Wu, Weiyan Zheng, Guoqing Wei, Enfan Zhang, He Huang, Jingsong He, Zhen Cai

**Affiliations:** ^1^Bone Marrow Transplantation Center, Department of Hematology, The First Affiliated Hospital, School of Medicine, Zhejiang University, Hangzhou, China; ^2^Institute of Hematology, Zhejiang University, Hangzhou, China

**Keywords:** multiple myeloma, maintenance, proteasome inhibitors, immunomodulators, optimal maintenance duration

There is an error in the title of the original article. The correct title is “Different Patient Subgroup Different Maintenance, Proteasome Inhibitors or Immunomodulators Maintenance for Newly Diagnosed Multiple Myeloma: A 7-Year Single-Center Data in China”.

The authors apologize for this error and state that this does not change the scientific conclusions of the article in any way. The original article has been updated.

## Publisher’s Note

All claims expressed in this article are solely those of the authors and do not necessarily represent those of their affiliated organizations, or those of the publisher, the editors and the reviewers. Any product that may be evaluated in this article, or claim that may be made by its manufacturer, is not guaranteed or endorsed by the publisher.

